# Hope, hype, and high costs: Navigating glucagon-like Peptide-1 based therapy initiation among young adults

**DOI:** 10.1016/j.obpill.2026.100338

**Published:** 2026-09-28

**Authors:** Sourav Das Gupta, Jaqueline Krystel Loubeau, Antara Swarnali Priyanka, Yajaira A. Cabrera Tineo, Janet Diaz-Martinez, Michelle M. Hospital, Mariana Sanchez, Rumi Agarwal

**Affiliations:** a Department of Health Promotion and Disease Prevention, Robert Stempel College of Public Health and Social Work, Florida International University, 11200 SW 8th Street, Miami, FL, 33199, USA; b Department of Epidemiology, Robert Stempel College of Public Health and Social Work, Florida International University, 11200 SW 8th Street, Miami, FL, 33199, USA; c Center for Research on US Latino HIV/AIDS and Drug Abuse (CRUSADA), Robert Stempel College of Public Health and Social Work, Florida International University, 11200 SW 8th Street, Miami, FL, 33199, USA; d Department of Dietetics and Nutrition, Robert Stempel College of Public Health and Social Work, Florida International University, 11200 SW 8th Street, Miami, FL, 33199, USA; e Community Based Research Institute, Florida International University, 11200 SW 8th Street, Miami, FL, 33199, USA; f Department of Biostatistics, Robert Stempel College of Public Health and Social Work, Florida International University, 11200 SW 8th Street, Miami, FL, 33199, USA

**Keywords:** Access barriers, Financial burden, Glucagon-like peptide-1 based therapy, Medication initiation, Weight management, Young adults

## Abstract

**Background:**

Glucagon-like peptide-1 (GLP-1)-based therapies have emerged as a major option for weight management, with prescribing among young adults increasing nearly sixfold between 2020 and 2023 in the United States. Despite this growth, little is known about how young adults decide to initiate GLP-1 therapy, navigate access, and manage treatment costs. This study examined individual, social, financial, and structural influences shaping GLP-1 initiation for weight management among young adults.

**Methods:**

This was a qualitative interview study that employed a semi-structured interview design utilizing a hybrid inductive-deductive thematic analysis framework guided by Andersen's Behavioral Model of Health Services Use. We recruited 25 young adults aged 18–30 years from the South Florida community who were currently using or had used GLP-1-based therapy for weight management within the preceding 12 months. Transcripts were coded deductively based on constructs from Andersen's model and inductively from themes identified directly from participant narratives.

**Results:**

Participants had a mean age of 22 years; 20 (80%) were female and 19 (76%) identified as Hispanic or Latino. Four themes were identified: (1) motivations and expectations for initiation, including health needs, appearance-related goals, and social media-shaped expectations; (2) social and clinical pathways to GLP-1 introduction, particularly family networks and social media; (3) navigation of access pathways ranging from conventional clinical care to medical spas, telehealth, and unregulated self-procurement; and (4) financial barriers characterized by out-of-pocket costs and insurance exclusions that contributed to informal procurement.

**Conclusions:**

Within this sample, GLP-1 initiation among young adults was shaped by intersecting health needs, social influences, access structures, and financial constraints rather than by clinical considerations alone. Findings highlight that healthcare systems, policymakers, and public health practitioners must prioritize clinically appropriate access, reliable medication sourcing, and consistent clinical oversight to promote equity and safety among young adults.

## Graphical abstract

**Figure:**
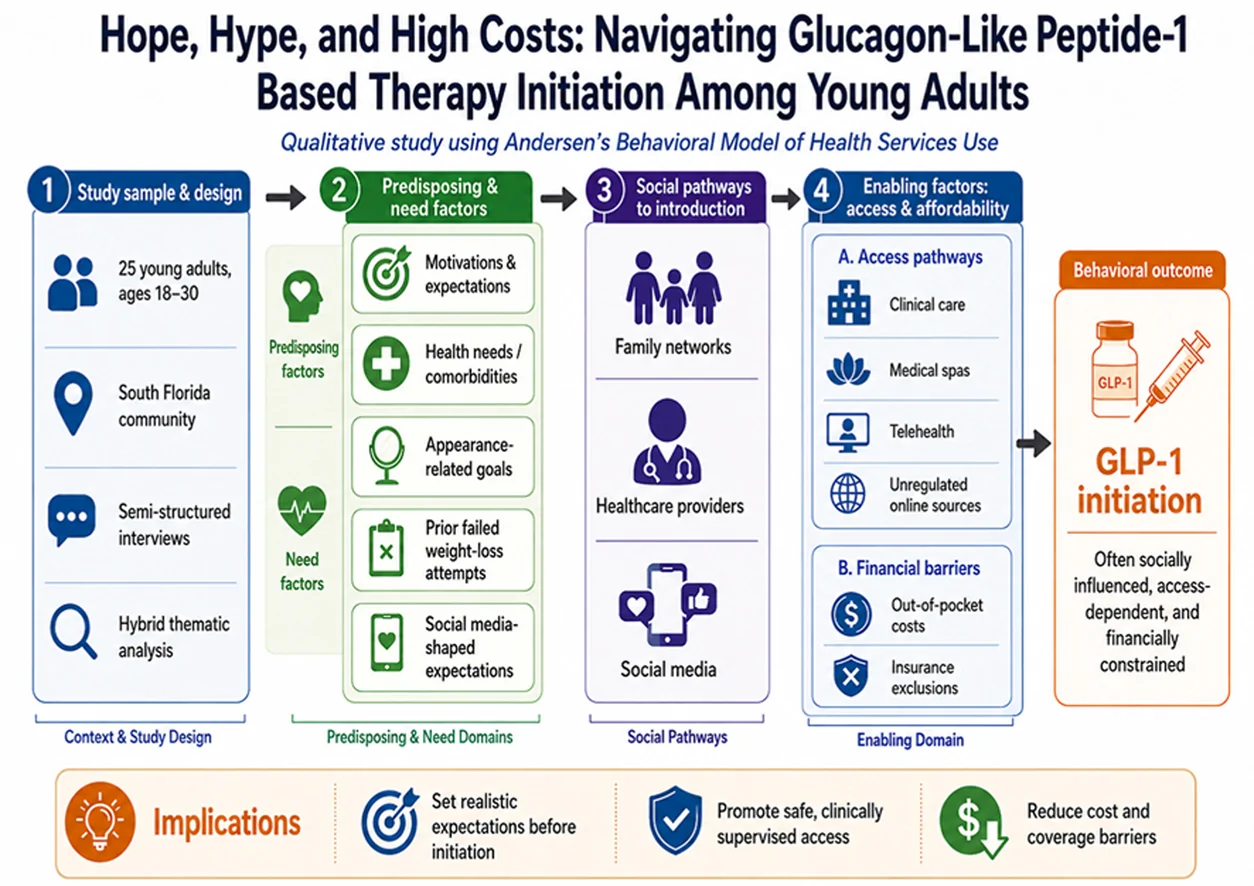

## 1 Introduction

Over the past three decades, the prevalence of obesity among young adults in the United States has roughly tripled [1,2], accompanied by a growing burden of type 2 diabetes [3,4]. Semaglutide and liraglutide are selective glucagon-like peptide-1 (GLP-1) receptor agonists, whereas tirzepatide is a dual glucose-dependent insulinotropic polypeptide (GIP)/GLP-1 receptor agonist. All of these therapies were developed for diabetes management, and growing evidence of efficacy has established this class of products as a major therapeutic option for weight loss as well as for glycemic control [[5], [6], [7]]. For readability, in this study we use GLP-1-based therapy, shortened to GLP-1, as an umbrella term for these agents, while recognizing that they are not pharmacologically interchangeable. Between 2020 and 2023, GLP-1 prescribing among adolescents and young adults in the U.S. increased nearly sixfold, with the largest increase observed among young adult women [8,9]. A Danish study further reported more than a 50-fold increase in GLP-1 use among adolescents and young adults aged 12–24 years old [10].

Despite this rapid growth, relatively little is known about how young adults make decisions about initiating GLP-1 medications for weight management. Young adults [defined as 18–30 years old] occupy a distinct biological, developmental, and socioeconomic position that differs meaningfully from adolescence and older adults [11,12]. Body image, peer influences, and social media may have particular relevance during young adulthood [13,14]. Unlike adolescents, young adults are legally positioned to make independent medical decisions and are more responsible for their own health [15]. They are also more likely to be uninsured and to lack a routine source of healthcare [16].

Predominantly, existing research is focused on GLP-1 initiation and utilization in middle-aged or older adults [[17], [18], [19]]. These studies document that adult decisions to initiate GLP-1-based therapy are shaped by intersecting health, psychosocial, and structural factors. Specifically, weight-related health concerns are identified as key drivers of treatment uptake [20,21], with one commonly reported benefit of relief from “food noise”, the persistent intrusive preoccupation with eating [20]. At the same time, concerns about side effects, injectable administration, dependence on medication, and weight regain following discontinuation play an important role [21]. Furthermore, perceived stigma and judgment stemming from pharmacologic weight loss [17] lead the psychosocial narrative, while structural determinants include access pathways, affordability, and insurance coverage [[22], [23], [24]].

It is unclear whether these findings from studies of middle-aged and older adults fully capture the experiences of young adults. To our knowledge, only one qualitative study has focused specifically on young adults, reporting that family influences, peer relationships, media exposure, appetite-related beliefs, and uncertainty regarding treatment duration all contributed to decision-making for GLP-1 initiation for weight loss [25]. While the study used the Health Belief Model [26] to capture individual perceptions of risk and benefit, it did not examine the structural and financial conditions, including pathways to care and insurance-related exclusions, that may enable or constrain young adults’ ability to act on those perceptions. Identifying these additional structural and financial determinants of GLP-1 initiation among young adults has significant implications for public health.

The current study aims to understand what combined influences and decision-making processes uniquely shape the commencement of GLP-1 for weight loss during young adults' lives. We draw on Andersen's Behavioral Model of Health Services Use [27], a well-established framework for understanding how individuals come to use health services that organizes determinants into three domains: predisposing, enabling, and need factors. Findings from this novel examination can explicitly inform policy and interventions to improve patient-centered care, shared decision-making between providers and patients, and equitable access to obesity treatment during a life stage that has important implications for long-term health outcomes.

## 2 Methods

### 2.1 Study design

The current study was a qualitative interview study that employed a semi-structured interview design utilizing a hybrid inductive-deductive thematic analysis framework guided by Andersen's Behavioral Model of Health Services Use [27]. We conducted 25 semi-structured individual interviews. The study was approved by the Florida International University Institutional Review Board (IRB-25-0570), and all participants provided electronic informed consent prior to data collection.

### 2.2 Setting and recruitment

Participants (N = 25) were recruited from the broader South Florida community through purposive sampling. Recruitment flyers were posted across university campuses, community centers, and gymnasiums, with a QR code linking to a REDCap screening form used to assess eligibility. Interviews were conducted via Zoom between February and April 2026; an in-person option was offered but not elected by any participant.

### 2.3 Eligibility criteria

Participants were eligible if they: (1) were between 18 and 30 years of age; (2) were currently using or had used a GLP-1 for weight management within the past 12 months, regardless of medication source; (3) were able to read and speak English fluently; and (4) were willing to participate in a 45–60-min individual interview. Participants were excluded if using GLP-1 solely for diabetes management, unable to complete the interview in English, or unwilling to provide informed consent.

### 2.4 Data collection

All interviews were conducted using a semi-structured guide covering medication initiation and motivation, access pathways, financial considerations, provider interactions, social influences, and expectations surrounding GLP-1 use, aligned with Andersen's constructs [27]. Interviews averaged approximately 40 min in duration. Interviews were audio-recorded and auto-transcribed; transcripts were reviewed for accuracy by team members prior to analysis.

### 2.5 Positionality statement

The research team comprised seven cisgender women and one cisgender man of Asian, Latino, and Haitian backgrounds. Several members have personal or familial experience with GLP-1 medications, providing familiarity with the clinical, social, financial, and emotional dimensions of participants' experiences. The primary researcher is a doctoral student in Health Promotion and Disease Prevention with a medical degree and substantial clinical experience, bringing both public health and clinical perspectives. Reflexive memo writing and peer debriefing were used throughout to examine assumptions and maintain grounding in participants' accounts.

### 2.6 Theoretical framework

Andersen's Behavioral Model of Health Services Use organizes the determinants of health service use into predisposing factors, which shape a person's propensity to seek care; enabling factors, the resources and structures that make care possible or constrain it; and need factors, both perceived and evaluated [27]. The model shaped this study at three points. It informed the domains covered in the interview guide, so that questions addressed access pathways, cost, and insurance alongside individual motivation. It supplied the deductive half of the codebook. It then provided the structure for organizing the four themes reported below, each mapped to its domain in Fig. 1. The model did not constrain inductive coding.

**Fig. 1:**
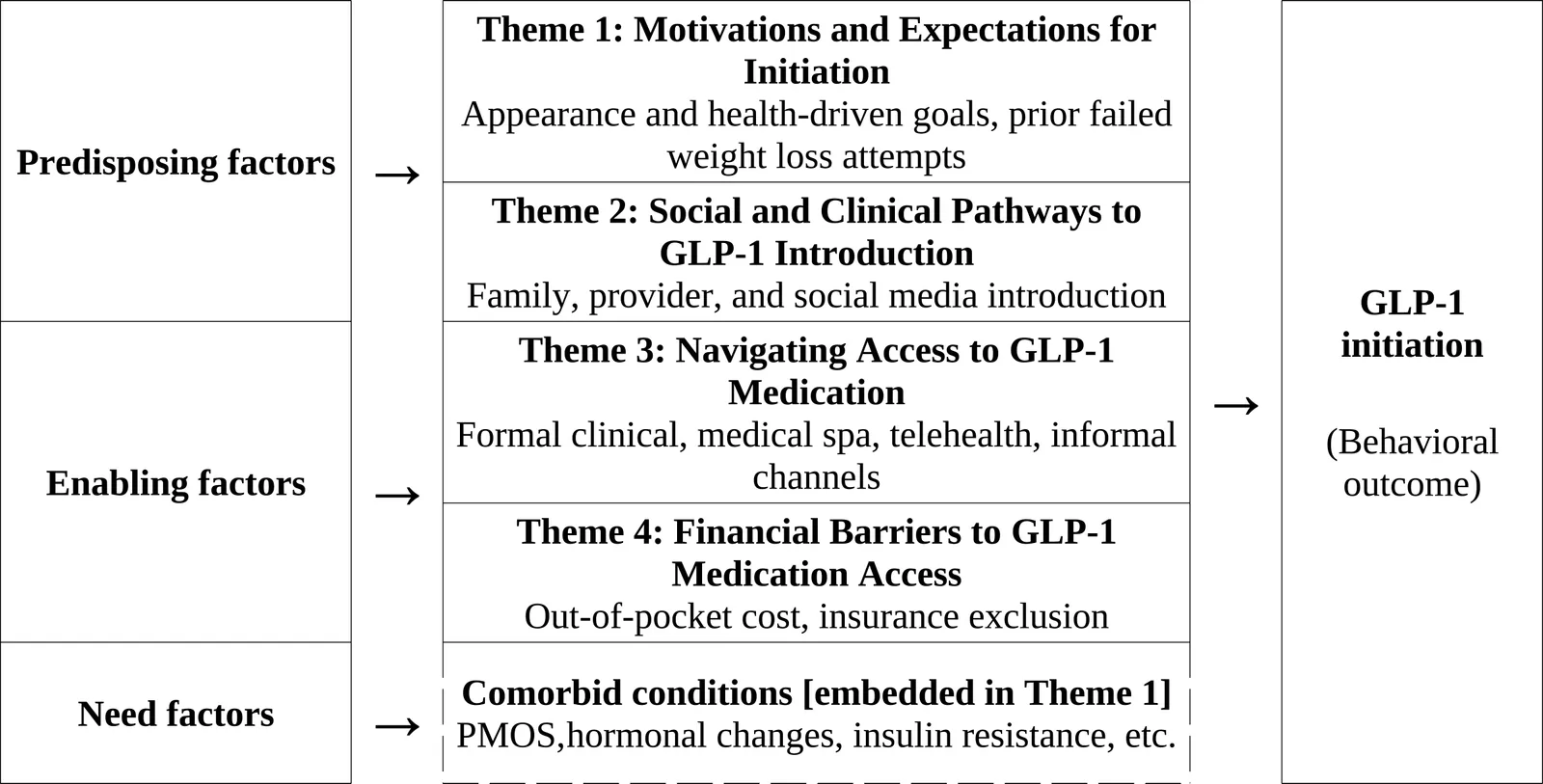
Study themes mapped onto the predisposing, enabling, and need domains of Andersen's Behavioral Model of Health Services Use. GLP-1, glucagon-like peptide-1; PMOS, polyendocrine metabolic ovarian syndrome, also known as polycystic ovary syndrome (PCOS); BMI, body mass index.

### 2.7 Data analysis

Interview transcripts were analyzed using a hybrid thematic analysis approach [28], which allowed data to be analyzed both deductively using predetermined codes derived from the major constructs of Andersen's Behavioral Model of Health Services Use [27], and inductively identifying additional codes directly from participant accounts.

The analytical process followed several iterative steps. First, the primary researcher and a team member independently reviewed seven transcripts and developed a working codebook through discussion. The primary researcher subsequently coded all 25 transcripts in NVivo 14 (QSR International), applying both deductive and inductive codes throughout. Another team member served as a peer debriefer, reviewing and challenging interpretations at regular intervals during the coding process. Reflexive memos were written by the primary researcher after each transcript to document analytical decisions, emerging insights, and reflexive observations. An audit trail was maintained to track all changes to the codebook across iterations. Together, these steps, including peer debriefing, reflexive memo writing, and maintenance of a transparent audit trail, were employed to enhance rigor and limit analytical bias [29]. Although thematic saturation was not used as a stopping criterion given the pilot sample size, the depth and consistency of themes across transcripts suggested adequate informational sufficiency.

## 3 Results

### 3.1 Sample description

The sample included 25 young adults aged 18–29 years, with a mean age of 22 years (SD = 2.9). Twenty participants (80.0%) were female and 19 (76.0%) identified as Hispanic or Latino, reflecting the demographic composition of South Florida, where the study was conducted. The largest group of participants was students, including 12 full-time students (48.0%) and 2 part-time students (8.0%). Nearly two-thirds reported annual personal incomes below $25,000, while the remaining third reported incomes ranging from $25,000 to $99,000 or preferred not to disclose. Fourteen participants (56.0%) were covered by parental insurance, followed by marketplace insurance (n = 5, 20.0%), employer-based insurance (n = 4, 16.0%), and student or Medicare coverage (n = 2, 8.0%). Participants reported the specific product used. Thirteen participants (52.0%) reported a branded product approved by the U.S. Food and Drug Administration (FDA): Zepbound (tirzepatide; n = 6, 24.0%), Ozempic (semaglutide; n = 5, 20.0%), Wegovy (semaglutide; n = 1, 4.0%), and Saxenda (liraglutide; n = 1, 4.0%). Eight of these participants (32.0%) reported a product approved for chronic weight management, and 5 (20.0%) reported Ozempic, which is approved for type 2 diabetes, indicating use outside the labeled indication because these participants did not have type 2 diabetes. The remaining 12 participants (48.0%) reported a compounded or otherwise unbranded formulation. We could not determine from participant report whether these products were obtained from a licensed pharmacy under clinical supervision or from an unregulated or unverifiable source, and we did not verify provenance independently. Compounded and unbranded formulations are therefore reported as a single participant-reported category, and procurement routes are described separately. Nineteen participants (76.0%) requested the medication rather than receiving a provider recommendation, a pattern of participants initiating contact with the healthcare system. Before initiating GLP-1, 24 participants (96.0%) had attempted exercise, and 23 (92.0%) had tried dietary modification for weight loss.

### 3.2 Thematic analysis findings

Four themes were identified. Comorbid health conditions, reflecting Andersen's need factors, are embedded within Theme 1 as a primary health-driven motivation for initiation, rather than constituting a standalone theme. All themes mapped onto Andersen's Behavioral Model (Fig. 1).

#### 3.2.1 Theme 1: motivations and expectations for initiation

Consistent with Andersen's predisposing factors, participants described multidimensional motivations for initiating GLP-1. Health-driven motivations, appearance-related goals, and prior failed weight loss attempts intersected to create a complex landscape. Expectations from the medication were often shaped by social narratives before any clinical contact.

Among the 25 participants, nearly half described a diagnosed or clinically suspected health condition that led to weight gain. For those participants, preventing further weight gain and weight-related complications was a primary driver for GLP-1 initiation. Polyendocrine metabolic ovarian syndrome (PMOS; previously termed polycystic ovary syndrome, PCOS) and weight gain related to birth control were frequently described as creating hormonal barriers that made conventional weight loss strategies harder despite sustained effort. Insulin resistance, pre-diabetes, high cholesterol, thyroid dysfunction, and elevated stroke risk were also reported. For several participants, the intersection of multiple comorbidities positioned GLP-1 medication as both a weight management tool and a preventive intervention for health complications. A 21-year-old Hispanic female participant said,

*“I also suffer from PCOS, so it's really hard for me to lose weight. I also have high testosterone levels and some problems with my thyroid, so that was another good reason why I wanted to start the shots and give myself that extra help."*

For a smaller subset, GLP-1 initiation followed a clinician's identification of elevated metabolic risk during a routine encounter. A 29-year-old Hispanic female participant stated,

*“I had to get a physical for work, and the doctor was looking at my blood work and asked me why I hadn't tried a medication to help with weight loss."*

Across all health-driven cases, prior engagement with conventional weight loss strategies was nearly universal. Twenty-four participants (96.0%) had tried exercise, and 23 (92.0%) had tried dietary modification before initiating GLP-1. Several participants described the medication as a last resort for themselves, adopted only after they felt they had exhausted other options. This reflects how participants perceived the hierarchy of treatment rather than a clinical position on where pharmacotherapy belongs in obesity care.

Several participants described appearance or self-image as a primary motivational driver. This was most evident among participants who described their own motivation as cosmetic or appearance-related rather than clinical reasons. As we did not collect body mass index (BMI) or other clinical data, we are unable to establish whether these participants met clinical eligibility criteria. An 18-year-old White female participant shared,

*“I was never overweight, and I do not have any health conditions. It was simply for cosmetic reasons."*

A few participants described event-based triggers, including prom, graduation, and vacation, that were associated with a sense of urgency around weight loss and medication initiation. A 21-year-old Hispanic female participant expressed,

*“I had a goal. I was trying to go to prom and look better, and I wanted to lose a couple of pounds."*

Fourteen of 25 participants described expectations at initiation. Weight loss was the primary expected outcome for nearly all participants, though the nature of that expectation varied considerably. A subset held realistic expectations about pace and effort, considering the medication as a supplementary tool alongside lifestyle change. A larger proportion entered with expectations shaped by social media representations of rapid transformation. A 22-year-old Hispanic female participant highlighted her expectations:

*“Honestly, I expected to have all my weight off in about a month. I think people believe it is going to produce a complete transformation quickly. I was expecting it to be like a magic pill."*

A smaller group also described expecting the medication to support a broader transition toward a healthier and more balanced lifestyle rather than weight loss alone.

#### 3.2.2 Theme 2: social and clinical pathways to GLP-1 introduction

Participants learned about GLP-1 through two primary channels: interpersonal networks (family members and healthcare providers) and social media. These pathways often functioned in combination, with initial introduction through one channel reinforced through another. Together, these reflect Andersen's predisposing factors that shaped participants' inclination to seek GLP-1.

Eleven of 25 participants first learned about GLP-1 medication through a family member, who had already used it with visible results. In several cases, the family member served simultaneously as the informational source, emotional motivator, and practical facilitator of access, connecting the participant to their own prescriber or clinic. A 25-year-old Hispanic female participant described:

*“My introduction to it was through family. My aunts and cousins were all on it and losing weight. I come from a heavier family, and when I saw it working for family members who do not even work out, I thought that if I added it to my exercise routine, it would be really beneficial. My aunt was the one who first suggested it, and my mom was supportive too. She actually offered to pay for it."*

Some participants were introduced by a healthcare provider, typically during a clinical encounter. A 21-year-old Hispanic female participant stated,

*“A doctor at [blinded institute], that's where they discovered I was having a stroke in my eye, because I had an inflamed optic nerve. The doctor there recommended the medication because she noticed I was very overweight. I'm only 5 feet tall, so it was very obvious. She recommended it first."*

Several participants identified social media as their primary introduction channel. Participants described repeated exposure to GLP-1 content through social media feeds on platforms like TikTok or Instagram featuring before-and-after transformations, influencer testimonials, or promotional advertisements. In several cases, participants described this exposure was enough to prompt them to seek the medication independently, on their own, without a clinical consultation. A 20-year-old Hispanic female participant described,

*“Social media is what first put this idea in my head. I would see girls posting that they're on something like ‘Freya’ which offers Ozempic or tirzepatide, showing their progress. That put the idea into my head and made me want it."*

Another 22-year-old Asian female participant said,

*“Every time I clicked on a related video, my feed would populate with more GLP-1 content. It caused an impulsive decision in me to buy it."*

Participant accounts also linked social media exposure to expectations about the speed and ease of weight loss. A 22-year-old Hispanic female participant described:

*“Social media portrays weight loss shots as miracle workers. You see the before and after, people look completely transformed, and it makes you think it is a fast and easy ticket to losing weight. That was convincing to me. It makes you think it is an easy route rather than something that requires real work alongside it."*

#### 3.2.3 Theme 3: navigating access to GLP-1 medication

All participants described accessing GLP-1 through pathways ranging from formal clinical evaluation to entirely unregulated self-procurement. This theme captures a core structural dimension of GLP-1 access and reflects Andersen's enabling factors, the resources, systems, and structures that facilitate or constrain health service use. Several participants obtained GLP-1 medication through a licensed healthcare provider that typically included medical history reviews, laboratory work, and clinical assessment of eligibility. A 23-year-old Hispanic female participant stated:

*“I spoke with an acute care nurse practitioner. She had me get blood work done, and once the results came back, she was able to tell me whether I was a candidate (for GLP-1 medication)."*

Several participants obtained medication through medical spas or weight loss clinics outside of conventional primary care providers. Participants reported differing levels of clinical evaluation and follow-up across these settings. Some described intake assessments that included body composition measurement and brief nutritional consultation, while others described minimal documentation before the medication was dispensed. A 21-year-old Hispanic female participant shared,

*“I went to a med spa type of location where they did some testing, urine, labs, things like that. Based on those results, they decided I was a good candidate and gave me the shots."*

A few participants accessed GLP-1 medication through telehealth applications or online pharmacies. These platforms ranged from structured telehealth services with assigned physicians to online ordering platforms. A 23-year-old Hispanic female participant shared,

*“I did it online through an app, which specializes in GLP-1 weight loss medication. They have a doctor assigned to you and you can message them almost around the clock."*

Apart from these access pathways, several participants obtained medication through informal or unregulated channels, including online marketplaces without a prescription requirement. A Hispanic 22-year-old female explained how she was able to order GLP-1 medication:

*“I searched for sites that let you order it online, then narrowed it down by price. I ended up going through a site. It wasn't through a primary doctor or telehealth, just directly through that site."*

#### 3.2.4 Theme 4: financial barriers to GLP-1 medication access

Financial burden was identified as a barrier to GLP-1 access across the sample, regardless of income level. The majority of the participants described paying for the medication entirely or largely out of pocket, and reported insurance exclusions across insurance types. This theme reflects Andersen's enabling factors as a constraint, where the absence of resources actively blocked or disrupted health service use. For more than half of the participants, who reported incomes below $25,000 or no personal income, this represented a substantial and ongoing financial strain. The burden was particularly acute for student participants, who frequently relied on parental financial support. A 20-year-old Hispanic female described,

*“I was trying to get a job and pay for everything. I used to have a job and saved up a lot of money to pay for my medication. And I lost my job, so, I pay out of pocket as I don't have insurance to cover it"*

Some participants explicitly described being denied insurance coverage for GLP-1 medication because they did not meet the qualifying diagnostic criteria. Participants described insurance exclusion as contributing to decisions to pay out of pocket and, in some cases, to seek less regulated access routes. A 20-year-old Hispanic female participant, who was under her parents’ insurance, described paying out of pocket after coverage was denied:

*“Staffs from my Doctor’s office initially tried to get my insurance to cover it, but since I don't have any comorbidities like diabetes or sleep apnea, they wouldn't cover it. So I just ended up paying out-of-pocket"*

Financial burden and insurance exclusion together formed an access barrier that was particularly pronounced for student participants, many of whom held marketplace or parental insurance plans under which they reported being denied coverage for GLP-1 for weight management. This coverage information came from participant report rather than from review of plan documents.

## 4 Discussion

The current study aimed to characterize the influences, access pathways, and financial circumstances shaping GLP-1 initiation for weight management among young adults, using Andersen's Behavioral Model of Health Services Use [27] to situate individual motivations alongside the structural and financial conditions. Four themes were identified: (1) multidimensional motivations and expectations for initiation, (2) social and clinical pathways to GLP-1 introduction, (3) navigation of a diverse access landscape, and (4) pervasive financial barriers to medication use. Taken together, findings revealed that GLP-1 initiation among young adults is shaped by a complex interplay of individual health needs, social influences, structural access barriers, and financial constraints. These findings both replicate and extend those of Nehushtan et al. (2026), the only prior qualitative study of GLP-1 initiation among younger adults [25]. Consistent with their findings, family influences and social networks emerged as central pathways for GLP-1 access. However, the present study extends beyond individual perceptions of risk and benefit to document the structural and financial determinants, including the spectrum of access pathways and insurance exclusion that shaped whether young adults could act on their decision to initiate.

### 4.1 Motivations reflect both clinical need and sociocultural influence

Motivations for GLP-1 initiation in the current sample were complex, consistent with Andersen's conceptualization of predisposing and need factors as encompassing both perceived and evaluated health needs [27]. While health-driven motivations or existing health conditions were prominent, appearance-related goals and event-driven triggers were equally present. Adult samples have similarly identified health-related concerns, including relief from ‘food noise,’ as a predominant driver of GLP-1 initiation [20]. By contrast, appearance-related goals and event-driven triggers were prominent motivators in this young adult sample, a pattern less emphasized in studies of middle-aged or older adults, and may reflect the heightened salience of body image and social comparison during this developmental stage [13,14]. This convergence of medical need and self-described cosmetic motivation in young adults may reflect the broader sociocultural context in which GLP-1 medications have rapidly become normalized.

Nearly all participants had previously tried to lose weight through conventional means. For most, GLP-1 initiation was a response to ongoing frustration from lack of results. This pattern is consistent with broader evidence that young adults with obesity face compounding physiological and psychosocial challenges in achieving sustained weight loss through lifestyle modification alone [20]. Several participants explicitly described the medication as a last resort for themselves, highlighting the persistent stigma and moral judgment surrounding pharmacological approaches to weight management. Participants reported requesting GLP-1 medication rather than having it recommended by a healthcare provider. This suggests that, for many participants, the healthcare visit functioned less as the point of treatment-seeking and more as a way to formalize a decision already made. This dynamic is consistent with emerging evidence from general practice settings, where clinicians have described patients as arriving “eager” and proactively booking appointments specifically to request these medications [30].

Participants’ medication expectations at initiation were further shaped by social media, a finding with important implications for consumer and provider communication. Specifically, a substantial proportion of participants expected a rapid transformation consistent with narratives prevalent on social media platforms. This misalignment between expectation and reality has been documented in the literature, as research has found that most GLP-1-related online content comes from non-medical creators and frequently misrepresents the pace, effort, and sustainability of weight loss outcomes [31,32]. No participants recalled expectation-setting from a clinician before starting, which points to a notable missed opportunity. Young adults should be clearly informed that GLP-1 medications are pharmacological tools, not standalone solutions, and that sustained outcomes require concurrent lifestyle engagement [33].

### 4.2 Social networks and digital media as primary introduction channels

Interpersonal networks, particularly family members, served as important sources of information for GLP-1 initiation. This pattern is consistent with evidence that social-network characteristics, including shared weight-control behaviors, supportive social norms, and social cohesion, may influence individuals’ intentions to engage in weight-management behaviors [34]. Family members in this sample served not only as sources of information but as facilitators of access. This transmission of health behavior is consistent with evidence that weight-related beliefs and practices can be transmitted across generations [35]. Given the predominantly Hispanic/Latino composition of the sample, the prominence of family involvement may also warrant further investigation in relation to culturally salient values such as familism [36], as cultural values were not directly assessed and cannot be confirmed from the current data.

Social media was described as a second introduction channel, and several participants said this exposure sometimes led to obtaining medication without consulting a clinician. This finding extends a growing body of evidence on the role of digital platforms in shaping GLP-1 utilization patterns. For example, a peer-reviewed study found that semaglutide (GLP-1) was frequently promoted on TikTok for off-label and appearance-related weight loss, often with limited information about indications and adverse effects, potentially contributing to misconceptions and uninformed use among followers [32]. Initiation influenced primarily by social media content, particularly in the absence of clinical evaluation, may increase the potential for inappropriate use, inadequate monitoring, or procurement through unregulated channels.

### 4.3 Access pathways span from formal care to unregulated self-procurement

A notable finding in the current study was the diversity of access pathways to GLP-1 medications. Participants described accessing medications through primary care physicians, endocrinologists, medical spas, telehealth platforms, online marketplaces, and informal social networks. This spectrum reflects the present-day environment regarding GLP-1 access in the US, in which rapidly increasing demand and supply constraints have created conditions that may encourage patients to seek medications through untraditional and potentially less regulated channels [37]. The IQVIA Institute for Human Data Science estimated that nearly 100 million prescriptions were dispensed through illegal online pharmacies in 2023, almost 50% more than in 2019, highlighting risks of counterfeit or adulterated products [37].

The greatest safety concerns arose among participants who described obtaining medications through informal channels without prior clinical evaluation, eligibility screening, or an established provider relationship. Some young adults also described initiating GLP-1 therapy for reasons they called cosmetic. The published literature raises concern that weight loss medication use among young people without a clinical indication may carry risk for disordered eating behaviors [38]. We did not assess eating behaviors and did not collect BMI, which is important context informed by the literature rather than a finding of the present study. Moreover, the absence of an established clinical relationship may reduce opportunities for systematic monitoring of adverse effects, contraindications, treatment response, and medication-related complications. Federal regulatory actions have begun addressing risks associated with unregulated GLP-1 products [39,40]. However, the diversity of pathways identified highlights the need for uniform standards and consumer guidance for young adults to navigate access with consistent safeguards.

The diversity of access pathways also reflects gaps in obesity care infrastructure. The World Health Organization's December 2025 guideline emphasized integrating GLP-1 into primary care-based chronic care platforms with trained providers, referral pathways, supply-chain capacity, behavioral support, and digital or telehealth tools for adherence and follow-up [41]. This vision stands in stark contrast to the fragmented, unsupervised access described by participants in this study. Consistent with Andersen's enabling factors framework, when formal enabling resources like accessible providers, covered medications, and navigable care systems are absent or inadequate, patients draw on alternative enabling structures [27]. These findings point to the importance of accessible, age-appropriate clinical counseling and reliable medication information for young adults.

### 4.4 Financial barriers compound access inequities and drive informal procurement

Financial burden emerged as a pervasive structural barrier to GLP-1 access across income levels and insurance types. Most participants reported paying entirely out of pocket for medication without insurance coverage. This burden was not merely inconvenient but potentially prohibitive. These findings are consistent with a nationwide survey, in which 68% of current or recent GLP-1 users said cost influenced their decision to initiate or continue therapy [42]. Although cost concerns are not unique to young adults, the financial burden reported in this sample may be proportionally greater given that most participants were early in their careers or still students, reliant on parental financial support to sustain treatment, a dependency less commonly observed in general adult GLP-1 cost-burden research.

Insurance exclusion compounded financial pressures. Several participants reported being denied coverage because they did not meet their insurer's eligibility requirements. This pattern reflects a structural misalignment between clinical eligibility guidelines and insurance coverage criteria. Furthermore, the insurance context in this sample differs from adult GLP-1 literature, which has largely focused on Medicare Part D coverage policy. Most participants in the current study reported insurance coverage through parental or employer plans that they described as excluding GLP-1 for weight management. This information came from participant report rather than review of plan documents, so we are unable to distinguish if plan-level exclusion from an individual denial was based on indication, BMI, comorbidity, prior authorization, or step therapy. Either way, it represents an understudied insurance gap affecting young adults specifically. Beyond individual insurance plans, broader coverage instability has also been documented at the state level, with some Medicaid programs dropping GLP-1 coverage due to high costs and unsuccessful pricing negotiations [43]. Although Medicare has historically excluded medications prescribed solely for weight loss from standard Part D coverage, the Centers for Medicare and Medicaid Services (CMS) launched the Medicare GLP-1 Bridge in July 2026. This short-term demonstration program, run through December 2027, provides eligible Part D beneficiaries access to selected GLP-1 medications for weight management at a standardized cost of $50 per month [44]. However, because Medicare eligibility primarily applies to adults aged 65 years or older and certain younger individuals who qualify through disability [45], this program offers limited direct benefit to the predominantly young adult.

Financial burden and insurance exclusion together raise a health equity concern: the conditions that would support safe, clinically supervised GLP-1 use were least available to the lowest-income participants in this sample. Whether broader coverage of obesity medications would reduce spending on obesity-related conditions is a question that should be investigated. While factors such as acquisition cost, duration of therapy as well as clinical effect are critical, a formal economic evaluation rather than inference from efficacy data is necessary. Addressing the access gap will require policy attention to coverage, standardized access requirements, and the costs participants described as contributing to their use of informal routes [46].

Two findings in this sample point in opposite directions and are worth holding together. Some participants described barriers to treatment they appeared to need, including denial of coverage in the presence of metabolic comorbidity. Others described obtaining medication with little or no clinical evaluation, in some cases for motivations they themselves called cosmetic. Under-access to indicated treatment and access outside clinical evaluation are not competing explanations of a single problem. They are two features of the same fragmented environment, and a policy response that addresses only one of them risks worsening the other.

### 4.5 Implications for practice and policy

Several implications follow from current findings. First, none of the participants recalled or described provider-mediated expectation-setting before initiation, suggesting a potential gap in counseling or in patients' retention of counseling. Initiation counseling is better understood as structured shared decision-making paired with appropriate clinical monitoring. It should cover indication and eligibility, the expected magnitude and trajectory of response, dose titration, common and clinically important adverse effects, contraindications and precautions, nutritional considerations, reproductive considerations where applicable, the expectation that treatment is likely to be long term, and the possibility of weight recurrence after discontinuation. Second, the prominence of social media as an introduction channel underscores the need for public health campaigns that provide accurate, accessible information about GLP-1 medications on platforms young adults use. Third, the variety of access pathways highlights the urgent need for regulatory frameworks that balance expanded access with consistent safety standards across all procurement routes. Finally, the pervasive financial barriers documented in this study reinforce the urgency of insurance coverage expansion for obesity medications, particularly for young adults on student or parental insurance plans for whom current exclusions create access gaps. Policy efforts should prioritize ensuring that young adults with clinical need have timely and affordable access to GLP-1 therapy, while also ensuring that access obtained outside clinical evaluation carries the safeguards that clinical evaluation provides.

### 4.6 Limitations

Several limitations apply. The sample was recruited from the South Florida community, was predominantly Hispanic or Latino, and included a substantial proportion of students, which limits transferability of the findings to other contexts. The study relied on self-report, which may be subject to recall bias or social desirability effects. Insurance coverage information came from participant report rather than from review of plan documents. We did not collect clinical data, including BMI, dosage, duration of use, side effects, or discontinuation history. Participants reported the product they used, which identifies branded products and, for those reporting Ozempic, use outside the labeled indication. It does not establish how many participants met clinical eligibility criteria for obesity pharmacotherapy, nor whether compounded products came from a licensed pharmacy under clinical supervision or from unregulated or counterfeit supply. Several findings therefore carry limited clinical interpretation. This constrains what can be concluded about appropriateness of use; however, it does not reduce the value of what participants reported about their own experience. With 25 participants, the study was designed for depth rather than for representativeness, and the findings are not offered as estimates of prevalence. A larger and more demographically varied sample is the priority for the next stage of this work. A longitudinal design would be needed to follow how access pathways and financial barriers change over the course of treatment.

## 5 Conclusions

The current study offers a qualitative analysis of young adults' perspectives and initiation experiences of GLP-1 therapy for weight loss, a population that sits at the center of the rapid expansion in GLP-1-based therapy use. Interpreted through Andersen's Behavioral Model of Health Services Use, findings reveal that predisposing factors, including health need, appearance goals, and social influence, intersect with enabling factors, including family networks, social media, access pathways, and financial resources, to shape a complex, often clinically unsupervised initiation process. Within this sample, initiation was socially embedded and frequently financially constrained, with some participants navigating informal access points. As GLP-1 use continues to expand, healthcare systems, policymakers, and public health practitioners must prioritize clinically appropriate access, reliable medication sourcing, and consistent clinical oversight to promote equity and safety among young adults.

## 6 Key messages

⁃ Young adults may initiate GLP-1 therapy through social and digital pathways before meaningful interaction with healthcare professionals, highlighting the need for early, structured clinical counseling.
⁃ Variation in access pathways, ranging from licensed clinicians to unregulated online marketplaces, underscores the importance of reliable medication sourcing and consistent clinical oversight.
⁃ Insurance exclusions and high out-of-pocket costs may contribute to young adults toward less regulated access routes, making affordable coverage an important component of safe obesity care.

## Ethical adherence and ethical review

This study was approved by the Florida International University Institutional Review Board (IRB-25-0570). All participants provided electronic informed consent prior to data collection. The study was conducted in accordance with the Declaration of Helsinki.

## Author contributions

Sourav Das Gupta: Conceptualization, Methodology, Investigation, Formal analysis, Validation, Writing – original draft;

Jaqueline Krystel Loubeau: Methodology, Validation, Writing – review & editing;

Antara Swarnali Priyanka: Methodology, Validation, Writing – review & editing;

Yajaira A. Cabrera Tineo: Writing – review & editing;

Janet Diaz-Martinez: Writing – review & editing;

Michelle M. Hospital: Writing – review & editing;

Mariana Sanchez: Writing – review & editing;

Rumi Agarwal: Conceptualization, Supervision, Writing – review & editing.

## Declaration of generative AI and AI-assisted technologies

During the preparation of this manuscript, the authors used Grammarly to improve sentence structure and language accuracy. Submission guidelines were checked by ChatGPT, and the graphical abstract was prepared with the help of ChatGPT and Claude. All AI-generated content was reviewed, critically evaluated, and edited by the authors. The authors take full responsibility for the integrity and accuracy of the content of this publication.

## Funding

This work was supported by 10.13039/100007681Florida International University through start-up funds provided to the senior author. The university had no role in the study design; collection, analysis, or interpretation of data; preparation of the manuscript; or decision to submit the article for publication.

## Declaration of competing interests

The authors declare no competing interests.
